# Homologous recombination repair is essential for repair of vosaroxin-induced DNA double-strand breaks

**DOI:** 10.18632/oncotarget.195

**Published:** 2010-11-22

**Authors:** Rachael Elizabeth Hawtin, David Elliot Stockett, Oi Kwan Wong, Cecilia Lundin, Thomas Helleday, Judith Ann Fox

**Affiliations:** ^1^ Sunesis Pharmaceuticals, Inc. 395 Oyster Point Boulevard, South San Francisco, CA 94080, USA; ^2^ Gray Institute for Radiation Oncology & Biology, University of Oxford. Old Road Campus Research Building, Roosevelt Drive. Oxford, OX3 7DQ, UK; ^3^ Dept. of Genetics Microbiology and Toxicology, Stockholm University. Arrhenius Laboratory, Svante Arrhenius väg 16 E4. S-106 91 Stockholm, Sweden

**Keywords:** DNA damage, homologous recombination, vosaroxin, topoisomerase II, cancer, subpopuations

## Abstract

Vosaroxin (formerly voreloxin) is a first-in-class anticancer quinolone derivative that intercalates DNA and inhibits topoisomerase II, inducing site-selective double-strand breaks (DSB), G2 arrest and apoptosis. Objective responses and complete remissions were observed in phase 2 studies of vosaroxin in patients with solid and hematologic malignancies, and responses were seen in patients whose cancers were resistant to anthracyclines. The quinolone-based scaffold differentiates vosaroxin from the anthracyclines and anthracenediones, broadly used DNA intercalating topoisomerase II poisons. Here we report that vosaroxin induces a cell cycle specific pattern of DNA damage and repair that is distinct from the anthracycline, doxorubicin. Both drugs stall replication and preferentially induce DNA damage in replicating cells, with damage in G2 / M > S >> G1. However, detectable replication fork collapse, as evidenced by DNA fragmentation and long tract recombination during S phase, is induced only by doxorubicin. Furthermore, vosaroxin induces less overall DNA fragmentation. Homologous recombination repair (HRR) is critical for recovery from DNA damage induced by both agents, identifying the potential to clinically exploit synthetic lethality.

## INTRODUCTION

Quinolone derivatives have recently been described as an alternative scaffold to the classic antineoplastic topoisomerase II poisons, including the anthracyclines, anthracenediones and epipodophyllotoxins [1-5]. These drugs are broadly used in the treatment of both solid and hematologic malignancies [6-8]. Objective responses and complete remissions in phase 2 studies of acute myeloid leukemia and platinum-resistant ovarian cancer were observed with vosaroxin (formerly voreloxin), a first-in-class anticancer quinolone derivative [9-11]. In both settings, responses were seen in patients whose cancers were resistant to anthracyclines. Vosaroxin is a DNA intercalating topoisomerase II poison [1], a feature it shares with the anthracyclines and anthracenediones. In contrast the epipodophyllotoxins do not intercalate DNA and directly interact with topoisomerase II.

Topoisomerase II exists in two isoforms, α and β, of which topoisomerase IIα has been studied most extensively. The enzyme is essential for the maintenance of DNA topology, disentangling DNA following replication, and is required to maintain correct chromosome condensation, decondensation, and segregation [12-14]. Expression of topoisomerase IIα is highest in mitotic cells and peaks at G2 / M phase of the cell cycle [12,15,16]. Hallmarks of topoisomerase II poisoning are the induction of DNA double-strand breaks (DSB) and G2 arrest [17]. However, the nature of the drug / enzyme / DNA interaction drives the specifics of DNA damage including sequence selectivity, location and extent of the induced DSBs, and the phase of the cell cycle in which they arise. Consequently the molecular characteristics of the drug-induced DNA damage and DNA damage response cannot be extrapolated directly from one molecular scaffold to another, based simply upon a common enzyme target. Thus the non-intercalating topoisomerase II poison etoposide causes extensive DNA laddering, while vosaroxin intercalates DNA and induces site-selective DNA DSB at G/C rich sequences [1], a characteristic of the quinolone core structure [18]. Further, although the anthracyclines are also DNA-intercalating topoisomerase II poisons, they drive additional DNA damage through non-topoisomerase II mediated mechanisms, including the induction of reactive oxygen species (ROS) [19,20]. The generation of ROS results in the formation of base mutations, anthracycline-DNA adducts and cross-links [21-26], and is linked to the scaffold-based cardiomyopathy associated with the anthracyclines [20,24,27,28]. These anthracycline-induced processes are not dependent upon the levels of expression of topoisomerase II, and the relative roles of each of them in the clinical activity and toxicity of the compounds is not fully established [8,20,22]. In contrast vosaroxin generates minimal ROS and the generation of ROS or DNA alkylation are not associated with the core quinolone structure [1].

Identification of the processes that repair vosaroxin-induced DNA DSB is critical for the further development of rational, clinically testable hypotheses that direct the selection of target indications and subpopulations. Two major pathways are active in the repair of DNA DSB; non-homologous end joining (NHEJ) and homologous recombination repair (HRR). The complexities of these pathways are reviewed by Wyman and Kanaar [29]. The phase of the cell cycle in which DNA damage is induced is critical in determining which of the response processes predominate. Because HRR requires a donor, homologous DNA sequence to replace the damaged region, this process is prevalent in mitotic cells when a copy of the target DNA is available for exchange [30]. The characteristics of the DNA damage further subdivide the molecular nature of the HRR response [31,32].

The focus of the current investigation was the analysis of the cell cycle phase specific toxicity of vosaroxin, and the identification of the DNA damage response processes that are critical to recovery from the associated toxic lesions. As a component of anthracycline toxicity is mediated through DNA intercalation and topoisomerase II poisoning, vosaroxin and doxorubicin were compared and contrasted in experiments that analyzed the extent and timing of DNA damage and cytotoxicity, and the DNA damage response mechanisms involved in repair of the damage. Understanding the processes that are essential for recovery from vosaroxin exposure will facilitate clinical exploitation of synthetic lethality.

## RESULTS

### Vosaroxin-induced DNA damage is preferential for replicating cells

**Figure 1:**
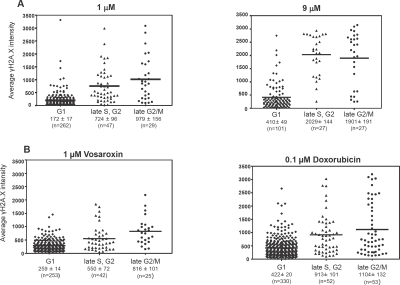
Vosaroxin- and doxorubicin -induced DNA DSB are increased in replicating cells The cell cycle dependence of vosaroxin-induced DNA damage was investigated using MO59K glioma cells which are large and highly adherent, and thus amenable to centrosome staining and analysis via immunofluorescence. Cells were treated for 6 hours with 1 or 9 μM vosaroxin (A) or 1 μM vosaroxin or 0.1 μM doxorubicin (B). Average γH2AX fluorescence intensity is displayed, with each symbol representing one cell. Cell cycle phases were established by analysis of centrosome size and number, and representative images are shown in Figure S1. The number of cells counted per phase is shown (n). Mean intensity is represented by horizontal line on the graph and listed below with standard error of the mean (SEM). Data are representative of three (vosaroxin) and two (doxorubicin) independent experiments.

The cell-cycle dependence of vosaroxin-induced DNA damage was investigated using MO59K glioma cells. Cell cycle phases were defined by centrosome size and number into G1 (single small centrosome), late S / G2 (larger centrosome) and late G2 / M (2 centrosomes) phases. Representative images are shown in Supplementary Figure 1. DNA damage was evaluated by staining for γH2AX and quantifying the stain intensity per nucleus, which is more diagnostic of DSB than overall γH2AX staining [33]. As shown in Figure 1, vosaroxin induced dose-dependent damage predominantly in the late G2 / M and late S / G2 populations. Vosaroxin at both 1 and 9 μM induced significantly less damage in G1 than in late S / G2 / M. In comparison with an equitoxic dose of doxorubicin (0.1 μM), vosaroxin induced less overall DNA damage.

### The vosaroxin-induced DNA fragmentation pattern differs from that of doxorubicin

**Figure 2:**
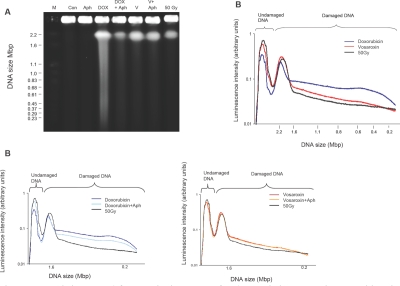
Vosaroxin induces DNA fragmentation independent of DNA synthesis, in contrast with doxorubicin which induces S phase dependent and independent fragmentation SPD8 cells were treated for 4 hr with 20 μM vosaroxin (V), 3 μM doxorubicin (DOX), 3 μM aphidicolin (Aph) or to vosaroxin or doxorubicin plus aphidicolin (+Aph) to arrest cells in S phase. Controls included 50 Gy of γ-irradiation (50 Gy) or DMSO only (Con). A) PFGE following 24 hr run. M = molecular markers. DNA fragment size is shown in Mbp. B) Luminescence intensity plots, in arbitrary units, showing data from vosaroxin treated cells +/− aphidicolin, doxorubicin treated cells +/− aphidicolin or an overlay of vosaroxin and doxorubicin treated cells with 50 Gy data included as positive control reference. A shift in the presence of aphidicolin indicates S phase dependent fragmentation. No difference was detected for vosaroxin +/− aphidicolin.

We have previously reported that vosaroxin induces dose-dependent and site-selective DNA fragmentation [1]. In the present study, the dependence of fragmentation upon active DNA synthesis was investigated and compared with doxorubicin. To ensure detectable DNA fragmentation, relatively high doses of vosaroxin (20 μM) and doxorubicin (3 μM) were used, with or without aphidicolin to induce an S phase block. SPD8 cells were exposed to drug or vehicle control for 4 hrs, prior to fragmentation analysis by pulsed-field gel electrophoresis (PFGE). Vosaroxin-induced DNA fragmentation appeared unaffected by aphidicolin (Figure 2). In contrast, doxorubicin induced a higher number of DNA fragments in the 1.6 – 0.2 Mbp range that were reduced in the presence of S phase block (Figure 2). A similar effect was previously reported for etoposide-induced DSBs [34].These data, combined with the DNA damage data shown in Figure 1, suggest that the vosaroxin-induced DNA fragmentation is distinct from that induced by doxorubicin and occurs during the G2 / M phases, while being undetectable during S phase.

### Vosaroxin and doxorubicin induce cytotoxicity both during and independent of S phase

**Figure 3:**
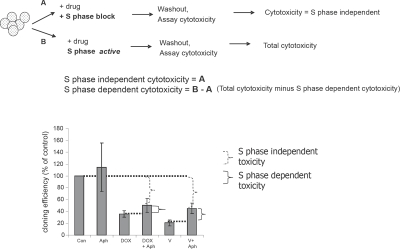
Vosaroxin and doxorubicin are cytotoxic both during and independent of DNA synthesis SPD8 cells were exposed for 4 hr to 2 μM vosaroxin (V), 0.3 μM doxorubicin (DOX), 0.5 μM aphidicolin (Aph), or with vosaroxin or doxorubicin plus aphidicolin (+Aph) to arrest cells in S phase. Controls were treated with DMSO only (Con). Colony growth was evaluated after 7 days. Cloning efficiency is plotted as percent of untreated control. The graphic outlines the experimental approach, which is designed to identify S phase dependent and independent cytotoxicity. S phase independent toxicity (dotted bracket) is identified by the growth inhibition that occurred in the presence of S phase block. The S phase dependent toxicity (solid bracket) is determined by subtraction of S phase independent toxicity from overall toxicity. Both drugs demonstrate a component of S phase toxicity, however the majority of cytotoxicity is S phase independent. Data represent the mean of 3 independent experiments, error bars represent SEM.

To determine when in the cell cycle vosaroxin-induced DSB are cytotoxic, SPD8 cells were exposed for 4 hours to equitoxic doses of vosaroxin or doxorubicin with or without aphidicolin, followed by 7 day colony growth in drug-free media. Although S phase independent toxicity (colony growth inhibition remaining in the presence of S phase block) accounted for the majority of growth inhibition by both drugs (Figure 3), a component of cytotoxicity was also S phase dependent.

### Vosaroxin-induced DNA damage is repaired by HRR processes that are cell cycle phase specific and are differentiated from doxorubicin

**Figure 4:**
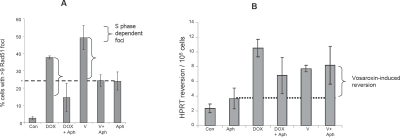
Vosaroxin and doxorubicin induce HRR during and independent of DNA synthesis A) Vosaroxin and doxorubicin trigger HRR during S phase. SPD8 cells were exposed for 4 hr to 2 μM vosaroxin (V), 0.3 μM doxorubicin (DOX), 0.5 μM aphidicolin (Aph), or with vosaroxin or doxorubicin plus aphidicolin (+Aph) to arrest cells in S phase. Controls were treated with DMSO only (C). The percent of cells with >9 RAD51 foci, representative of HRR activation, is plotted. The dashed horizontal line represents RAD51 foci induced by aphidicolin alone. Brackets represent the differential between vosaroxin or doxorubicin +/− aphidicolin (ie; vosaroxin alone minus vosaroxin + Aph). A decrease in the presence of Aph for both drugs indicates S phase dependent induction of RAD 51 foci. Data represent the mean of 3 independent experiments, error bars represent SEM. B) S phase block reduces doxorubicin-induced but not vosaroxin-induced long tract recombination. SPD8 cells were exposed for 4 hr to 2 μM vosaroxin (V), 0.3 μM doxorubicin (DOX), 0.5 μM aphidicolin (Aph), or with vosaroxin or doxorubicin plus aphidicolin (+Aph) to arrest cells in S phase. Controls were treated with DMSO only (C). The same population of treated cells were plated for cloning efficiency (Figure 3). Revertants / recombinants were selected by growth for 7 days in the presence of HAsT. The horizontal dashed line denotes reversion / recombination in the presence of aphicicolin control. To determine S phase dependent recombination events that are induced by each drug, the number of recombination events in the presence of drug + Aph are subtracted from the number of events with drug alone (ie; revertants with vosaroxin alone minus vosaroxin + Aph). No significant difference was detected for vosaroxin. A modest but significant difference was detected for doxorubicin (p=0.04). Data are plotted as revertants / 10^5^ cells, and represent the mean of 3 independent experiments. Error bars represent SEM.

HRR has been shown previously to be involved in the repair of doxorubicin-induced DNA damage [23] and plays a major role in DNA DSB repair during S and G2 / M phases of the cell cycle (reviewed by Helleday et al [30]). To further elucidate vosaroxin's molecular mechanism of action, and to identify cellular backgrounds which may be particularly sensitive to the drug, the contribution of HRR to the repair of vosaroxin-induced DNA damage was investigated and compared with doxorubicin. Both the activation of HRR and the implementation of long tract recombination were assessed. HRR activation was detected by analysis of RAD51 focus formation, which serves as an early HRR signal with broad substrate specificity [3,4]. As shown in Figure 4A, exposure for 4 hr to either doxorubicin or vosaroxin triggered RAD51 foci formation. The blockade of DNA synthesis reduced the number of foci to levels comparable with (vosaroxin) or below (doxorubicin) the level of aphidicolin control, indicating that the detected foci represent HRR triggered during active DNA synthesis. Consistent with these data is the dose-dependent S phase prolongation induced by vosaroxin (Supplementary Figure 2). In contrast, vosaroxin-induced long tract recombination, as detected by *hprt* reversion, was not detectably reduced by S phase block (Figure 4B), suggesting that HR-mediated reversion events occur principally if not exclusively at G2 / M. Contrasting with vosaroxin, doxorubicin-induced recombination events were modestly but significantly reduced by S phase block (p = 0.04), indicating that long tract recombination contributes to the repair of doxorubicin-induced DNA damage both during and independent of DNA synthesis. The moderate level of reversion events that were induced by both drugs is representative of topoisomerase II targeting agents [34].

### HRR compromised cells are sensitized to vosaroxin and doxorubicin

**Figure 5:**
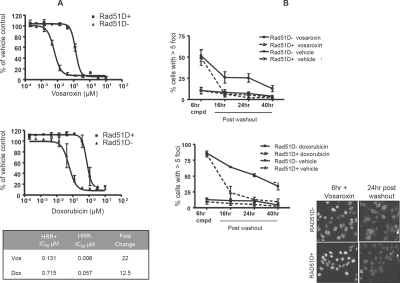
HRR compromised cells are sensitized to vosaroxin and doxorubicin A) Vosaroxin and doxorubicin are more cytotoxic for RAD51D null cells. The matched cell lines RAD51D1 (RAD51D null) and RAD51D1.3 (matched, RAD51D reconstituted) were exposed to a dose-titration of vosaroxin, doxorubicin or DMSO control for 72 hr. Inhibition of proliferation is plotted as the percent of control. The mean IC_50_'s for each drug in both cell lines, and the fold difference in sensitivity between lines, is tabulated. Loss of RAD51D increased sensitivity to both drugs. Data represent the mean of two independent experiments, error bars represent SEM. B) HRR compromised cells are unable to completely repair vosaroxin- and doxorubicin-induced DNA damage. RAD51D1 and RAD51D1.3 cells were treated for 6 hr with 0.11 μM vosaroxin, 1 μM doxorubicin, or with DMSO control, followed by washout and temporal evaluation of recovery from DNA damage, via quantification of γH2AX foci. DNA damage was evaluated upon compound removal (at 6 hr) and 16, 24 and 40 hr after washout. The graphs show the percent of cells at each time point with >5 γH2AX foci. Repair of DNA damage was compromised following treatment with each drug. Data represent the mean of 2 independent experiments, error bars represent SEM. Representative images are shown for each cell line at the time of compound removal and 24 hr following washout.

The CHO AA8 RAD51D mutant cell line (clone 51D1) harbors a genetic knockout of RAD51D and shows increased sensitivity to DNA DSB-inducing agents, while the matched line (clone 51D1.3) is reconstituted for RAD51D expression [35]. These cell lines were used to examine the role of HRR in recovery from vosaroxin and doxorubicin-induced cytotoxicity. As shown in Figure 5A, cells with a compromised HRR pathway were 22-fold more sensitive to vosaroxin-induced inhibition of proliferation, and 12.5-fold more sensitive to doxorubicin. Further, as shown in Supplementary Figure 3, increased sensitivity to vosaroxin-induced G2 arrest was observed in the HRR compromised mutant background. Onset of arrest was observed at 0.004 μM as compared to 0.037 μM (approximately 10-fold shift) in the HRR competent cell line.

To confirm that the enhanced sensitivity of HRR compromised cells is a function of reduced ability to repair vosaroxin- or doxorubicin- induced DNA damage, DNA repair was evaluated over time following 6 hr exposure to the compounds. Cells were treated with equitoxic doses of vosaroxin, doxorubicin or with DMSO control, followed by washout and quantification of RAD51 foci over time. As shown in Figure 5B, within 16 hr the number of detectable foci in vosaroxin- or doxorubicin-treated HRR competent cells was reduced to levels comparable with DMSO-treated controls, whereas HRR compromised cells sustained levels of foci that were significantly above background levels for the duration of the assay (40 hr). Thus the enhanced sensitivity to both vosaroxin or doxorubicin of HRR compromised cells correlates with an impaired ability to repair drug-induced DNA DSB.

### BRCA2 deficiency sensitizes cells to vosaroxin and doxorubicin

**Figure 6:**
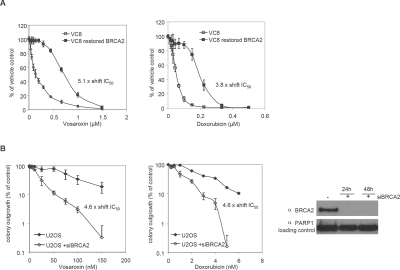
BRCA2 loss sensitizes cells to vosaroxin and doxorubicin A) Cells expressing truncated BRCA2 are sensitized to vosaroxin more than doxorubicin. VC8 (mutant BRCA2) and VC8-B2 (restored BRCA2) cells were treated for 4 hr with a dose-titration of vosaroxin, doxorubicin, or with DMSO control. Inhibition of proliferation was evaluated following 5 days incubation and is represented as percent of DMSO control. Graphs represent the mean of 2 independent experiments and error bars represent SEM. The shift in IC_50_ between cell lines is shown for each compound. B) BRCA2 knockdown sensitizes cells equally to vosaroxin and doxorubicin. siRNA knockdown of BRCA2 was performed in U20S human sarcoma cells, followed by treatment with a dose-titration of vosaroxin or doxorubicin, or with DMSO control. Colony growth inhibition was evaluated following 14 days incubation and is graphed relative to DMSO control. Error bars represent SEM. The efficiency of knockdown at 24 and 48 hr post transfection, with BRCA2 siRNA or transfection agent alone (-) is shown. The shift in IC_50_ between conditions is shown for each compound.

The role of BRCA2 in HRR, and the established synthetic lethality of molecules targeting DNA damage and repair in the BRCA2 mutant background [36-38], prompted the analysis of vosaroxin sensitivity in CHO cell lines mutant (V-C8) and competent for BRCA2 (V-C8B2, BRCA2 reconsitituted) [39]. BRCA2 mutation sensitized cells to inhibition of proliferation by both vosaroxin (5.1-fold) and, in keeping with data reported by Spencer et al [23], doxorubicin (3.8 fold) (Figure 6A). Further, in the U20S human sarcoma cell line, siRNA knockdown of BRCA2 induced a 4.6-fold sensitization to colony growth inhibition by both vosaroxin and doxorubicin (Figure 6B). Thus the cytotoxicity of both agents is influenced to a comparable extent by the functionality of the HRR pathway, despite differential induction of HRR-mediated recombination events during DNA synthesis.

## DISCUSSION

The studies reported here provide molecular detail of the mechanism of action of vosaroxin, the first of a new class of antineoplastic agents, the anticancer quinolone derivatives. These data establish that vosaroxin-induced DNA DSB are preferential for replicating cells. Consistent with the interdependence of DNA repair mechanisms, DNA replication and cell cycle checkpoints [40,41], the mechanisms invoked to repair these DSB differ with cell cycle phase. Further, several points of differentiation from the classic topoisomerase II poison, doxorubicin, were identified, largely occurring during the S phase of the cell cycle.

The relative induction of damage induced during G2 / M > S >> G1 is consistent with previous reports that analyze the cell cycle phase of DNA DSB induction by topoisomerase II targeting agents [42], and with the peak in expression of topoisomerase IIα at the G2 phase of the cell cycle [7,12,13,15]. Both vosaroxin and doxorubicin induced DSB during G2 / M, as established by detectable DNA fragmentation and consequent HRR-mediated long tract recombination. Consistent with these observations, the majority of vosaroxin and doxorubicin-induced cytotoxicity occurred independent of S phase, indicating that the DSB induced during G2 / M are the principal cause of cytotoxicity.

Points of contrast between vosaroxin and doxorubicin were identified in their S phase-induced DNA damage and associated DNA damage responses. Allthough vosaroxin's S phase-induced DNA damage triggered the HRR response, as detected by RAD51 focus formation, this DNA damage was not associated with detectable DSBs or recombination. This indicates that replication fork collapse is absent in vosaroxin-treated cells, or occurs below the level of detection. Vosaroxin did induce a dose-dependent S phase delay, suggesting that cleavable complexes form a replication fork barrier, consistent with previously reported data [43]. Contrasting with vosaroxin, a greater number of DNA fragments (0.2 – 1.6 Mbp) were induced by doxorubicin, which also caused replication fork collapse as evidenced by detectable levels of DNA fragmentation and long tract recombination during S phase.

**Figure 7:**
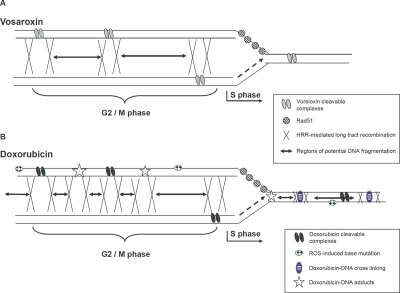
Model for induction of differential, cell cycle phase specific DNA damage and HRR processes following exposure to vosaroxin or doxorubicin DNA in G2 / M phase is shown on the left, with the replication fork advancing during S phase to the right. Points of homologous recombination are depicted by X, with the region exchanged depicted by the horizontal arrow. These regions also represent potential DNA fragments in the absence of DNA repair. A) Vosaroxin-induced DNA damage is depicted in the upper model, where the replication fork is stalled due to topologic stress in DNA, resulting in a prolonged S phase and the induction of RAD51 foci. Replication fork collapse is either below detectable levels or is absent, evidenced by undetectable DNA fragmentation or HRR-mediated long tract recombination during S phase. The toxicity of S phase induced lesions is low. In G2 / M phase, DNA DSB induction is maximal, inducing detectable DNA fragmentation, HRR-mediated long tract recombination and maximal toxicity. B) Doxorubicin-induced DNA damage is depicted in the lower model, where in addition to cleavable complexes base mutations, DNA cross-linking and DNA adducts are also generated. The advancing replication fork encounters torsional stress in the DNA and RAD51 foci are induced. A proportion of active replication forks collide with DNA lesions (shown here as a DNA adduct), inducing DNA fragmentation (represented by the horizontal arrows) and long tract recombination, indicating replication fork collapse. Sites for additional potential fork collapse are shown by the X, in regions of doxorubicin-DNA cross links and adducts. Fewer DNA DSB are formed in S phase than in G2 / M and cytotoxicity of the lesions is lower. In G2 / M phase, DNA DSB induction is maximal, resulting in increased DNA fragmentation, HRR-mediated long tract recombination and maximal toxicity. Because of the increased number and / or diversity of DNA interactions, DNA DSB induction and fragmentation is greater than that induced by vosaroxin, so generating an increased number of smaller (1.6 – 0.2 Mbp) fragments. This is represented by the increased number of, and smaller size of, the horizontal arrows.

These data are summarized in a model in which vosaroxin induces two major forms of toxic DNA lesion that are repaired by HRR, and are generated during different phases of the cell cycle (Figure 7A). During DNA synthesis in vosaroxin treated cells, the replication fork may encounter torsional stress due to cleavage complexes in proximity to the sites of DNA replication. The HRR response is activated and localized to the region of the replication fork which stalls, slowing DNA synthesis. The advancing fork does not collide with the lesion or collapse, thus DNA fragmentation and long tract recombination are undetectable and the toxicity of these lesions is low. During G2 / M, where the cell is actively dividing and when topoisomerase IIα expression is elevated, vosaroxin induces an increased number of topoisomerase II-mediated DSB and detectable DNA fragmentation, maximizing cytotoxicity during this phase. Long tract repair via HR contributes to cellular recovery from the damage.

Replication forks collapse when they collide with regions of damaged DNA, and when DNA repair processes are unable to stabilize or re-start stalled or blocked forks [40,44]. The combined effects of disparity in both number and mode of molecular interactions with DNA, as a consequence of their contrasting chemical structures [1], is a plausible explanation for the differences in the DNA damage and HRR induced by vosaroxin and doxorubicin, and is represented as a model in Figure 7B. Although both drugs intercalate DNA and poison topoisomerase II, the anthracyclines induce DNA damage through additional, non topoisomerase II-mediated mechanisms [20,22], including the induction of ROS. Anthracycline-induced ROS generate mutagenic base modifications [19,45] and drive the formation of additional bulky lesions in the form of DNA adducts and crosslinks [21-26], lesions which cause replication fork stall [40,46] and collapse [47]. HR plays a role in the repair of these forms of damage [23,47,48], and the DSB caused by interstrand crosslinks are in particular associated with the S phase of the cell cycle [49]. Following doxorubicin treatment these diverse drug / DNA interactions may induce enough S phase DNA damage and / or generate a particularly toxic form of lesion(s), to drive the observed replication fork collapse. The contributions of the different anthracycline- DNA interactions to cytotoxicity of the drugs are as yet unresolved [20,22]. In contrast vosaroxin produces minimal ROS [1] and the generation of ROS or DNA alkylation are not characteristics of the quinolone core. Thus vosaroxin may produce fewer bulky lesions in S phase, predominantly in the form of topoisomerase II cleavage complexes, a consequence of which is minimal or absent replication fork collapse. The overall reduction in DNA fragmentation, relative to doxorubicin, may also be attributable to these contrasts in molecular reactivity. Despite these molecular mechanistic differences, vosaroxin induced S phase dependent toxicity that was comparable with doxorubicin. This may be due to a requirement for cells to pass through S phase and reach G2 / M phases where vosaroxin activity is maximal.

The increased vosaroxin sensitivity of HRR mutant cells forms the basis of a clinically testable hypothesis, exploiting synthetic lethality to target identifiable subpopulations which may be particularly sensitive to the drug. These include tumors harboring mutations in BRCA1 and 2, which impair HRR and increase sensitivity to drugs that target DNA repair pathways [36-38,50]. Indications harboring such mutations include breast (particularly triple negative breast cancer), prostate and ovarian cancers [50-53].

Collectively, the data reported here establish a cell cycle phase specific pattern of vosaroxin-induced DNA damage and fragmentation, and the reflection of these in the induction of critical, phase-specific HRR response processes. Points of contrast between vosaroxin and doxorubicin in the extent and timing of DNA fragmentation, and the phase-specific HRR processes that are induced, highlight molecular mechanistic divergence between these structurally unrelated topoisomaerase II poisons. These mechanistic observations are critical to the generation of clinically testable hypotheses that drive rational drug development.

## MATERIALS AND METHODS

### Cells and cell culture

MO59K, A549 and U20S cell lines were obtained from American Type Culture Collection. RAD51D1 and RAD51D1.3 Chinese Hamster Ovarian (CHO) matched clones were the kind gift of Dr Lawrence Thompson, Lawrence Livermore Research Laboratories, CA. The CHO cell lines SPD8, VC8 and VC8B2 have been previously described [38,54].

A549, MO59K and RAD51D matched clones were cultured and maintained at 37°C and 5% CO_2_ atmosphere in RPMI-1640 media supplemented with 10% fetal bovine serum (Cellgro). U20S and VC8 cell lines were cultured and maintained in Dulbecco's Modified Eagle's Medium (DMEM), with the addition of 9% fetal calf serum and penicillin-streptomycin (90 U/mL) at 37°C and 5% CO_2_ atmosphere. VC8-B2 cells were cultured in DMEM as above, with the addition of G418 (Sigma Aldrich) to 100 μg/mL. SPD8 cells were cultured in DMEM containing 9% fetal calf serum, penicillin-streptomycin and 6-Thioguanine (6TG, 5 μg/mL; Sigma Aldrich) in order to kill cells that undergo spontaneous reversion.

The SPD8 cell line contains an inactivating partial duplication of the hprt gene that serves as an endogenous readout for HRR; a functional gene is regenerated through reversion mediated by long tract HRR [54]. These cells were therefore used to evaluate the induction of HR-mediated reversion following treatment with compound. The same cells were used for compound-induced DNA fragmentation, cytotoxicity and HRR activation experiments, allowing for the evaluation of the HRR response in a consistent cellular background.

The CHO AA8 RAD51D mutant cell line (clone 51D1) harbors a genetic knockout of RAD51D and shows increased sensitivity to DNA DSB-inducing agents, while the matched line (clone 51D1.3) is reconstituted for RAD51D expression [35]. These cells were used to examine the drug sensitivity of HRR compromised cells.

The VC8 cell line has a truncating mutation in the *brca2* gene and VC8-B2 is this cell line complemented with the human chromosome 13 (containing the *brca2* gene) [39]. These cells were used to evaluate the role of BRCA2 in recovery from compound-induced DNA damage.

### Reagents

Aphidicolin (Sigma Aldrich), doxorubicin (Sigma Aldrich) and vosaroxin (Sunesis Pharmaceuticals) were dissolved in dimethylsulfoxide (DMSO) to a maximal final DMSO concentration of 0.2%.

### Immunofluorescence analyses

Detection of γH2AX and pericentrin foci in MO59K cells:

MO59K glioma cells are large, highly adherent and thus amenable to centrosome staining and analysis via immunofluorescence. Cells were seeded overnight at 10,000 / well in 96-well plates, followed by 6 hr treatment with compound. At 6 hr cells were fixed, permeabilized and stained for γH2AX and pericentrin as follows. Cells were washed in PBS and fixed for 10 mins with 0.2% paraformaldehyde in PBS. Paraformaldehyde was removed and the cells permeabilized for 5 mins with 0.5% TritonX-100, then washed with PBS and fixed with 2% paraformaldehyde in PBS for 10 mins. Following a PBS wash, cells were blocked for 1 hr with 5% BSA in PBS and incubated overnight at 4°C with primary antibodies (see below). Cells were washed with PBS and incubated in the dark for 1 hr at room temperature in secondary antibodies (see below). Cells were then washed in PBS and nuclei stained by adding 1 ug/ml Hoechst 33342 in PBS to each of the wells. The cells were analyzed using an ArrayScan high content screening device. Nuclei were identified as objects based on the Hoechst 33342 stain.

γH2AX staining: γH2AX was detected using mouse anti- γH2AX (Upstate 05-636) diluted 1:500 in 1% BSA in PBS. Secondary antibody, AlexaFluor 594-conjugated goat anti-mouse (Invitrogen A31623), was diluted 1:300 in 1% BSA in PBS. For MO59K cells, the mean γH2AX intensity was measured for each nucleus. For RAD51D1 and D1.3 cells γH2AX foci were counted within each nucleus, and a cell was considered to be positive for γH2AX if the nucleus contained >5 foci.

Pericentrin staining: Pericentrin was detected using rabbit anti-pericentrin (Abcam; ab4448) diluted to 1:1000 in 1% BSA in PBS. Secondary antibody, AlexaFluor 488-conjugated anti-rabbit IgG secondary antibody (Invitrogen, A11008), was diluted 1:1000 in 1% BSA in PBS. Cells were assigned to cell cycle phases as follows: small single nucleus, G1; larger, more diffuse nucleus, late S / G2; two nuclei, M.

Detection of γH2AX foci inRAD51D CHO cells:

RAD51D1 and RAD51D1.3 cells were seeded overnight at 10,000 / well in 96-well plates, followed by 6 hr treatment with compound or DMSO control diluted in growth media. Each treatment was performed in duplicate. Cells were then washed with fresh media and grown in the absence of compound. At washout and 16, 24, and 40 hr post washout the cells were fixed, permeabilized, and stained for nuclei and γH2AX as described above.

Detection of RAD51 foci in SPD8 cells:

Cells were seeded overnight at 8,000 / well in 96-well plates, followed by 4 hr treatment with vosaroxin or doxorubicin +/− aphidicolin, aphidicolin alone or with DMSO control diluted in growth media. Cells were fixed for 20 min in 4% paraformaldehyde in PBS-T (PBS + 0.1% Triton X-100), washed in PBS, permeabilized with PBS + 0.3% Triton X-100 and blocked for 40 mins with 3% BSA in PBS. Cells were incubated overnight at 4 °C with rabbit anti-RAD51 (Santa Cruz Biotechnology, H-92) diluted 1:1000 in 3% BSA in PBS. Following PBS wash cells were labeled for 1 hr, at room temperature in the dark, with alexa 488-conjugated goat anti-rabbit (Invitrogen, A31629) diluted 1:500 in 3% BSA. After PBS wash, cells were stained for 5 mins at room temperature with 50 μL DAPI (1 μg/mL), washed and analyzed using an INCell Analyzer 1000 (GE Healthcare). Images were analysed using the INCell Analyzer 1000 Workstation software (GE Healthcare), counting at least 300 nuclei per treatment condition. Each treatment was performed in sextuplicte or septuplicate. A cell was considered as positive for RAD51 foci if it contained >9 foci.

### Pulsed-Field Gel Electrophoresis (PFGE)

SPD8 cells were seeded overnight at 2×10^6^ /75 cm^2^ flasks, followed by 4 hr treatment with vosaroxin or doxorubicin +/− aphidicolin. Cells were washed with PBS before being melted into agarose inserts (1×10^6^ cells/70 μL 1% InCert Agarose, BMA), cooled at 4 °C and placed in sarcosyl solution (1% N-laurylsarcosyl, 1 mg/mL proteinase K, 0.5 M EDTA pH 8.0) at 50°C for 48 hours. Control inserts were irradiated with 50 Gy γ-irradiation in a Cs137 chamber (1.9 Gy/min) prior to incubation in sarcosyl solution. Inserts were rinsed 4 times for 2 hrs in TE and loaded into the wells of a 1% Chromosomal grade agarose gel (BioRad). Separation was performed on a CHEF DR III (BioRad; 120°, field switch 60–240 sec, 4 V/cm) for 24 hr at 14 °C. The gel was stained with ethidium bromide for 5 hours and subsequently analyzed by scanning fluorescence reader (Molecular Imager FX, Biorad) using Quantative One software.

### Colony growth inhibition

SPD8 cells were seeded at 1.5 × 10^6^/75 cm^2^ flask and incubated 24 hr prior to 4 hr treatment with vosaroxin, doxorubicin or DMSO control +/− aphidicolin. Compound was washed out and cells incubated for 48 hrs, then plated in duplicate in 10 cm dishes at 500 cells / dish (or seeded for reversion assay analysis as described below). After 7 day incubation the plates were harvested and the colonies fixed and stained using methylene blue in methanol (4 g/L). Colonies containing more than 50 cells were counted.

### Reversion assay

SPD8 cells were seeded and treated as described above for analysis of colony growth inhibition, thus cytotoxicity and reversion are established from the same population of treated cells. For reversion analysis, following drug exposure, washout and recovery, cells were seeded in triplicates at 3 × 10^5^ cells / dish in the presence of HAsT (50 μM hypoxanthine,10 μM L-azaserine, 5 μM thymidine) to select for revertants to wild type hprt, and incubated for 10 days. Plates were harvested and the colonies fixed and stained using methylene blue in methanol (4 g/L). Colonies containing more than 50 cells were counted.

### Measurement of RAD51D1 and RAD51D1.3 proliferation by MTT

RAD51D1 and RAD51D1.3 cells were plated and grown overnight in 96-well plates at 2000 cells / well and treated (in duplicates) with a dose-titration of vosaroxin, or with DMSO control, for 72 hr. After treatment, MTT reagent (5 mg/mL, Sigma-Aldrich) was added directly to the media and incubated at 37°C/5% CO_2_ for 2 h. MTT lysis buffer was added and cells were incubated at 37°C/5% CO_2_ overnight. Samples were analyzed by measuring the light absorbance at 595 nm using a SpectraMax plate reader (Molecular Devices). Values obtained for treatment samples were normalized to control samples.

### Measurement of VC8 and VC8-B2 proliferation by resazurin

VC8 and VC8-B2 cells were plated and grown overnight in 96-well plates at 4000 cells/well and treated for 4 hr (in duplicate) with serial dilution of drug or with vehicle control. Following PBS rinse cells were incubated for 5 days before staining with resazurin (10μg/mL DMEM). Fluorescence was measured using the Envision plate reader (Ex530nm/Em590nm, Perkin Elmer) and growth inhibition plotted as percentage fluorescence compared to untreated cells.

### siRNA knockdown and U20S Colony growth inhibition

U20S human sarcoma cells were seeded in 6 well plates, 2 × 10^5^ / well, and incubated overnight. Cells were transfected with 100 pmol siBRCA2 (siGenome SMARTpool, 5′-GAAACGGACUUGCUAUUUA-3′, 5′-GUAAAGAAAUGCAGAAUUC-3′, 5′-GGUAUCAGAUGCUUCAUUA-3′, 5′-GAAGAAUGCAGGUUUAAUA-3′ Dharmacon) mixed with 2 μL DharmaFect 1 (Dharmacon), or with transfection reagent alone, in a total volume of 2 mL antibiotic free media. Following 24 hr incubation cells were trypsinized, counted and seeded into 10 cm plates at 500 or 1000 cells / plate. After 4 hr incubation cells were treated with a dose-titration of vosaroxin or doxorubicin and incubated for 14 days. Colonies were fixed and stained using methylene blue as described above.

### Western blot

Cell lysates were separated on a 3–8% Tris-Acetate gel, transferred to nitrocellulose membrane, the membrane cut to allow dual analysis of target and control antigen and incubated overnight at 4°C in either mouse anti-PARP (Santa Cruz Biotechnology, clone PARP-1 (F-2) or mouse anti-BRCA2 (Calbiochem, OP95) antibody, diluted 1:500 in 5% milk-TBST. Membranes were washed in TBST, incubated for 1 hr at room temperature with HRP-conjugated anti-mouse antibody (Millipore, AP501P), washed and developed using chemiluminescence (Roche).

## SUPPLEMENTAL FIGURES

Supplemental Figure 1

Supplemental Figure 2

Supplemental Figure 3
